# Prediction and prevention of ventilation impairments during bronchoscopy

**DOI:** 10.1186/s40635-025-00846-5

**Published:** 2025-12-17

**Authors:** Ben Fabry, Navid Bonakdar, Christian Kuster, Johannes Bartl, Frederick Krischke, Roland Francis

**Affiliations:** 1 Department of Physics, Friedrich-Alexander Universität Erlangen-Nürnberg, Henkestr. 91, 91052 Erlangen, Germany; 2 Department of Anesthesiology, Universitätsklinikum Erlangen, Friedrich-Alexander Universität Erlangen-Nürnberg, Erlangen, Germany

**Keywords:** Mechanical ventilation, Endotracheal tube resistance, Bronchoscopy, Intrinsic PEEP, Hypoventilation

## Abstract

**Background:**

Bronchoscopy in ventilated patients narrows the endotracheal tube lumen and increases resistance, which can lead to hypoventilation and intrinsic PEEP build-up. These ventilation impairments depend on the geometry of the tube–bronchoscope combination, ventilator settings, and patient mechanics. Currently, no predictive method exists to quantify these impairments or guide compensatory strategies.

**Methods:**

We measured pressure–flow relationships across multiple tube–bronchoscope configurations in a bench setup and derived a scaling law describing the nonlinear, flow-dependent resistance as a function of the effective tube diameter, defined as the diameter of a circular tube with the same open cross-sectional area as the remaining lumen. We then assessed the ventilatory consequences of bronchoscopy using an intensive care ventilator connected to an active lung simulator under both volume- and pressure-controlled modes.

**Results:**

Bronchoscope insertion sharply increases resistance, which scales with the inverse fifth power of the effective diameter. A simulation tool based on this scaling law accurately predicts the experimentally observed dynamic hyperinflation and intrinsic PEEP build-up in volume-controlled modes, as well as the reduced tidal volumes in pressure-controlled modes. Ventilation with automatic tube compensation during pressure control fully prevents both impairments.

**Conclusions:**

The commonly cited recommendation of a ≥ 2 mm difference between endotracheal tube and bronchoscope diameters does not reliably prevent ventilation impairments during bronchoscopy. Our findings suggest that a quantitative framework, which accounts for ventilator settings, patient mechanics, and the effective tube diameter, can provide additional guidance for tube selection and help anticipate impairments. We demonstrate proof of principle that pressure-controlled ventilation with automatic tube compensation is a feasible strategy to mitigate bronchoscopy-induced ventilation impairments.

**Supplementary Information:**

The online version contains supplementary material available at 10.1186/s40635-025-00846-5.

## Introduction

More than 30% of intensive care patients require mechanical ventilation, most commonly delivered invasively via endotracheal intubation [1–3]. Intubation, however, is associated with adverse effects, such as mucosal injury, impaired mucociliary clearance, and an increased risk of bacterial colonization of the lungs [4, 5]. In addition, the endotracheal tube introduces a large, nonlinear resistance and causes airflow limitations [6, 7]. As a result, depending on the tube’s inner diameter, dynamic hyperinflation and intrinsic positive end-expiratory pressure (iPEEP) build-up may develop during controlled mechanical ventilation [7, 8]. Moreover, the tube resistance is a major contributor to increased work of breathing and patient–ventilator asynchrony in spontaneously breathing patients [9, 10].

Airflow limitations are further amplified when a bronchoscope is inserted through the endotracheal tube, which substantially decreases the available lumen. Flexible bronchoscopy is a frequently performed diagnostic and therapeutic procedure in mechanically ventilated intensive care patients [11]. Common indications include evaluation of persistent infiltrates, removal of mucus plugs, and bronchoalveolar lavage [12].

Alterations in airflow dynamics during bronchoscopy can be substantial, especially for combinations of small endotracheal tubes and large bronchoscopes. In such cases, the intrinsic PEEP build-up in volume-controlled modes and the reduction of tidal volume in pressure-controlled modes can reach critical levels [13, 14]. However, these effects are also dependent on ventilator settings and the patient’s respiratory system elastance [13, 14]. Consequently, continuous bedside estimation of iPEEP and tidal volume is mandatory during bronchoscopy, but remains technically challenging and prone to error. Compensation strategies that dynamically adjust airway pressure to account for increased tube resistance, such as automatic tube compensation (ATC), may offer a potential solution, although they have not yet been evaluated during bronchoscopic procedures.

The aim of this bench study is to provide a practical, quantitative framework for predicting and minimizing ventilatory impairments during bronchoscopy. To this end, we characterize the nonlinear flow-dependent resistance of tube–bronchoscope combinations and develop a software tool for predicting iPEEP and tidal volume as a function of ventilator settings and patient respiratory mechanics. In addition, we test if the mode automatic tube compensation can prevent ventilation impairments during bronchoscopy.

## Methods

**Fig. 1:**
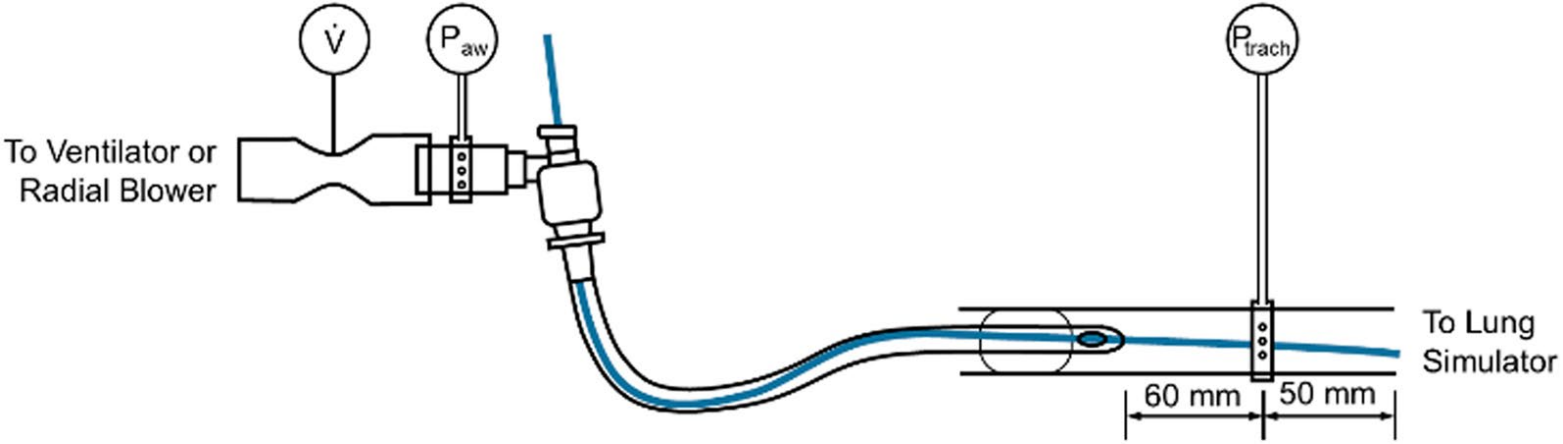
Experimental setup for measuring the influence of a bronchoscope (blue) on endotracheal tube resistance, airflow, and pressure patterns during mechanical ventilation. The endotracheal tube is inserted into a 2.1 cm inner diameter Plexiglas tube serving as an “artificial trachea.” The bronchoscope is advanced through the endotracheal tube beyond the tracheal pressure measurement site

For measuring the resistance of original-length endotracheal tubes (Rüsch Super Safety Clear, Teleflex, Ireland; inner diameter 6–9 mm in 0.5 mm increments), we replicated the setup described in [6] (Fig. 1). In brief, we inserted the endotracheal tube and cuffed it airtight in an artificial trachea (Plexiglas tube) with an inner diameter of 2.1 cm. Tracheal pressure was measured through 12 small, equally spaced radial holes positioned around the circumference of the artificial trachea, located 60 mm distal to the tip of the endotracheal tube and 50 mm proximal to the end of the artificial trachea (Fig. 1). Similarly, airway pressure was measured through 8 small, equally spaced radial holes in a tube placed between the swivel connector and the flow meter.

Airway and tracheal pressures were measured with piezo-resistive differential pressure sensors (HCS-series Honeywell (USA) sensors, ± 80 mbar range). Gas flow was measured with a factory-calibrated thermal mass flow sensor (SFM3300, Sensirion, Switzerland). Flow and pressure signals were sampled at 250 Hz and digitally low-pass filtered using a fourth-order Bessel filter with a cutoff frequency of 25 Hz.

The artificial trachea was connected to a custom-built active lung simulator consisting of a 1.8 L syringe with a piston driven by a stepper motor via a spindle-nut mechanism. The simulator applies the equation of motion of the respiratory system at an update rate of 250 Hz (see SI for more details). Respiratory system compliance was set to 50 ml/mbar and airway resistance to 2 mbar/(L/s). Muscular pressure was set to zero to emulate the absence of spontaneous breathing efforts.

For measuring the tube resistance, the lung simulator was removed, and a radial blower (U65HN-024KS-6, Micronel, Switzerland) was connected to the flow meter. The motor speed of the radial blower was slowly ramped up and down to generate a maximum pressure (at zero flow) of + 80 mbar (at the blower outlet), or -80 mbar (at the blower inlet). Equation 1 was fitted to the measured pressure-flow relationship across the tube using least-squares optimization implemented in the SciPy library of Python.

For exploring the pressure and flow patterns during pressure- and volume-controlled mechanical ventilation, we used an intensive care ventilator (EVITA V600, Dräger, Germany) in combination with the lung simulator. Ventilator settings during volume-controlled ventilation were: tidal volume V_T_ = 500 ml, positive end-expiratory pressure PEEP = 0, inspiratory time T_in_ = 1.8 s without end-inspiratory pause, expiratory time T_ex_ = 2.2 s. Parameters during pressure-controlled ventilation were: pressure support = 10 mbar above PEEP, PEEP = 0, T_in_ = 1.8 s, T_ex_ = 2.2 s, pressure ramp time = 0.

To deliver pressure-controlled mechanical ventilation with automatic tube compensation (ATC), we used a prototype ventilator based on the system described in [10], with the following modifications: positive and negative pressures were generated by two radial blowers (U65HN-024KS-6, Micronel, Switzerland) connected to the inspiratory and expiratory limbs of a Y-piece, respectively. The patient limb of the Y-piece was then connected to the flow meter (Fig. 1). Pressure was controlled by a three-way valve integrated directly into the Y-piece, as described in [15]. Ventilator settings were as follows: PEEP = 0 mbar, T_in_ = 1.8 s, T_ex_ = 2.2 s. Over the course of the inspiratory phase, the target tracheal pressure was linearly ramped up from 0 to 10 mbar, and then linearly ramped down during expiration.

We performed measurements with single-use bronchoscopes (aScope 4 Broncho, Ambu, Ballerup, Denmark) in three sizes—small, medium, and large—with measured shaft diameters of 3.8 mm, 5.0 mm, and 5.9 mm, respectively. Bronchoscopes were introduced into the endotracheal tube via the swivel connector cap through a perforated silicone membrane seal. To prevent air leakage during use of the smallest bronchoscope, the membrane opening was additionally sealed with adhesive putty (Patafix, Bolton Adhesives). We measured each experimental condition or combination once, as the measurements are reproducible and yield deterministic pressure–flow relationships.

Tidal volume was computed by numerically integrating the measured flow during inspiration. Intrinsic PEEP was estimated breath-by-breath from the end-expiratory tracheal pressure, which is a close proxy for the alveolar pressure under the conditions of a small end-expiratory gas flow and a small airway resistance of 2 mbar/(L/s).

Numerical simulations of flow and pressure patterns during pressure-controlled and volume-controlled ventilation were performed using a custom Python script, freely available under the MIT license at https://github.com/fabrylab/Bronchoscopy. This repository also includes a browser-based version of the simulation program, implemented in HTML and JavaScript, which can be downloaded and run locally in a web browser, or accessed directly via https://fabrylab.github.io/Bronchoscopy/.

## Results

### Resistance of the endotracheal tube

**Fig. 2:**
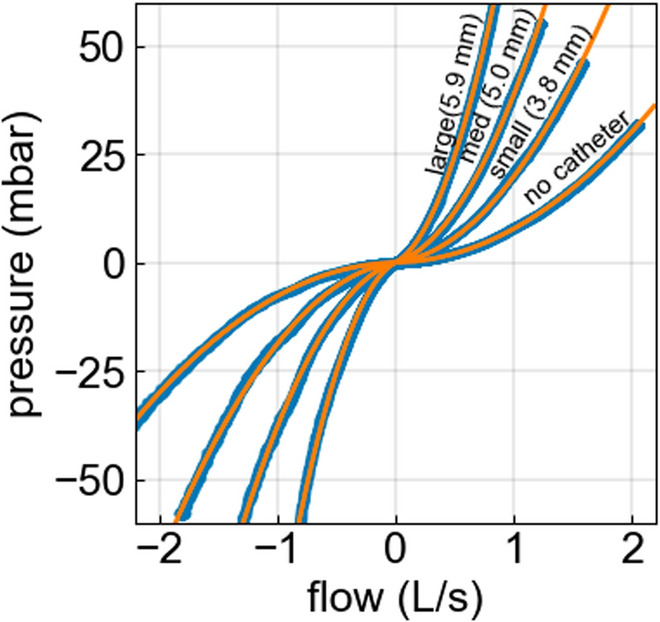
Pressure–flow relationship of an 8 mm inner diameter endotracheal tube before and after inserting bronchoscopes with different outer diameters. Blue dots are measured values; orange lines represent the fit of Eq. 1 to the data

The pressure difference across the endotracheal tube Δp_ETT_, measured as the difference between the airway pressure p_aw_ and the tracheal pressure p_trach_ (Fig. 1) shows a strong nonlinear increase with gas flow, $\dot{\text{V}}$ (Fig. 2). This nonlinear pressure versus flow relationship is well-captured by the Rohrer equation with two free parameters, k1 and k2 [6, 16, 17] (Fig. 1):

1 $${\Delta \text{p}}_{\text{ETT}}=\text{k}1 \dot{\text{V}}+\text{sign}(\dot{\text{V}}){\text{k}2\left(\frac{\dot{\text{V}}}{{\dot{\text{V}}}_{0}}\right)}^{2}$$

${\dot{\text{V}}}_{0}$ is a reference flow, which we set to 1 L/s, so that k1 + k2 corresponds to the total pressure drop at a flow of 1 L/s. k1 describes the fraction of the total pressure drop across the tube that grows linearly with flow, and k2 describes the fraction that grows quadratically with flow. k1 is considered to be the same in inspiration and expiration, while k2 can be different in inspiration and expiration due to the compression–expansion asymmetry of the flow at the transition between the tip of the endotracheal tube and the trachea [6]. Using separate k2 parameters in inspiration and expiration slightly improves the fit of Eq. 1 to the data for a given tube–bronchoscope combination. When comparing different tube–bronchoscope sizes and combinations; however, k2 does not systematically differ between inspiration and expiration (Table S1). Values for k1 and k2 for all combinations of endotracheal tube diameters and bronchoscope diameters are given in Table S1, with k2 being listed separately for inspiration and expiration.

**Fig. 3:**
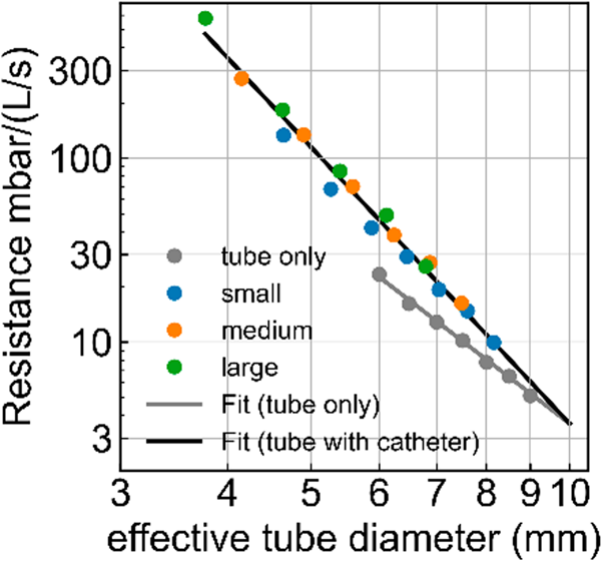
Double-logarithmic plot of tube resistance (pressure drop across the tube measured at a flow of 1 L/s, as taken from the Rohrer parameters k1 + k2 (Eq. 1) averaged for inspiration and expiration), as a function of the effective tube diameter D_eff_. Points indicate measured values, the gray line indicates the fit R = 3.6 (D_eff_/D_0_)^−3.6^, and the black line the fit R = 3.6 (D_eff_/D_0_)^−5^, with reference tube diameter D_0_ set to 10 mm

In the following, when we refer to numerical values of resistance R, we specifically mean the secant resistance of the tube at a flow of 1 L/s (the pressure drop at 1 L/s divided by the flow), and hence we report the numerical values of R = k1 + k2, averaged for inspiration and expiration, in units of mbar/(L/s). We find that in the absence of a bronchoscope, the resistance of endotracheal tubes increases with decreasing inner tube diameter D according to a power-law relationship, R ~ D^−3.6^ (Fig. 3, gray points and line).

### Tube resistance during bronchoscopy

Inserting a bronchoscope into an endotracheal tube dramatically increases the resistance (Fig. 2), as indicated by an increase both of the linear and the quadratic resistance parameters k1 and k2 (Fig. 3, Table S1). Instead of following an inverse power-law relationship with exponent -3.6, however, the resistance increases more steeply with decreasing effective diameter D_eff_ according to R ~ D_eff_^−5^ (Fig. 3), where D_eff_ is the diameter of a circular tube with the same cross section as the tube–bronchoscope combination:

2 $${\text{D}}_{\text{eff}}=\sqrt{{{\text{D}}_{\text{tube}}}^{2}-{{\text{D}}_{\text{scope}}}^{2}}$$

${\text{D}}_{\text{tube}}$ is the internal diameter of the endotracheal tube, and ${\text{D}}_{\text{scope}}$ is the outer measured shaft diameter (not the nominal diameter provided by the manufacturer) of the bronchoscope. Table 1 lists the effective diameters of all tube–bronchoscope combinations investigated in this study.

**Table 1:** Effective inner diameters (mm) of endotracheal tubes with and without bronchoscope

	Bronchoscope sizes		
ETT ID	Small	Medium	Large
(mm)	(3.8 mm)	(5.0 mm)	(5.9 mm)
9	8.2	7.5	6.8
8.5	7.6	6.9	6.1
8	7.0	6.3	5.4
7.5	6.5	5.7	4.6
7	5.9	5.0	3.8
6.5	5.3	4.3	–
6	4.6	–	–

*ETT ID* = endotracheal tube internal diameter

### Effects of tube resistance on dynamic hyperinflation during volume-controlled ventilation

**Fig. 4:**
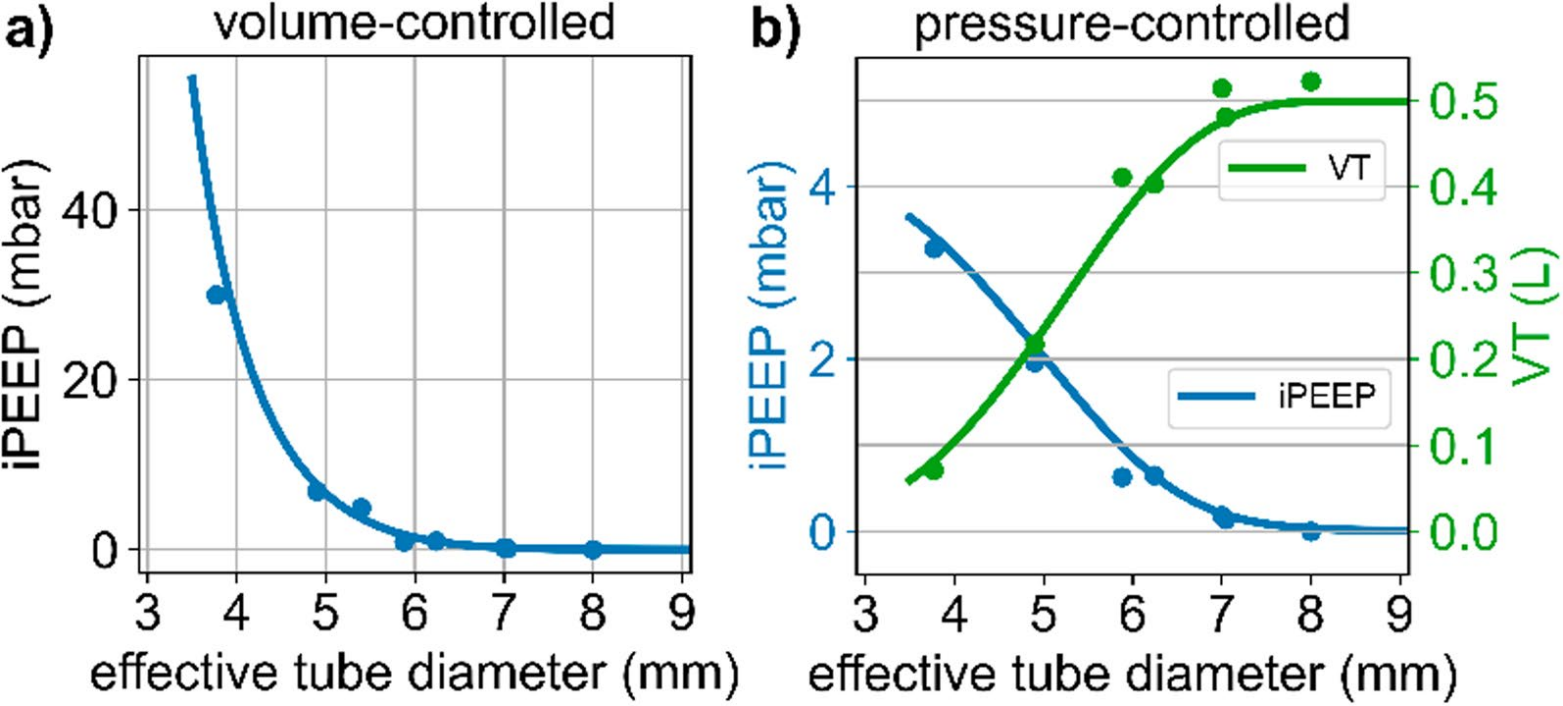
**a** Intrinsic PEEP build-up during volume-controlled mechanical ventilation, for V_T_ = 500 ml, T_in_ = 1.8 s and T_ex_ = 2.2 s, as a function of the effective tube diameter. **b** Intrinsic PEEP build-up (blue) and tidal volumes (green) during pressure-controlled mechanical ventilation for a pressure support of 10 mbar, T_in_ = 1.8 s and T_ex_ = 2.2 s, as a function of the effective tube diameter. In both graphs, the lines show the results from a numerical simulation based on the resistances of tubes with inserted bronchoscope taken from the fit shown in Fig. 3 (black). Dots are measured values obtained with an EVITA V600 ventilator and lung simulator (C = 50 ml/mbar, R_aw_ = 2 mbar/(L/s) for different tube–bronchoscope combinations

A numerical simulation of the flow conditions during volume-controlled mechanical ventilation predicts that intrinsic PEEP increases sharply as the effective tube diameter falls below 5 mm, even for a rather moderate situation with relatively low tidal volume (500 ml) and low respiratory rate (15/min) (Fig. 4a).

**Fig. 5:**
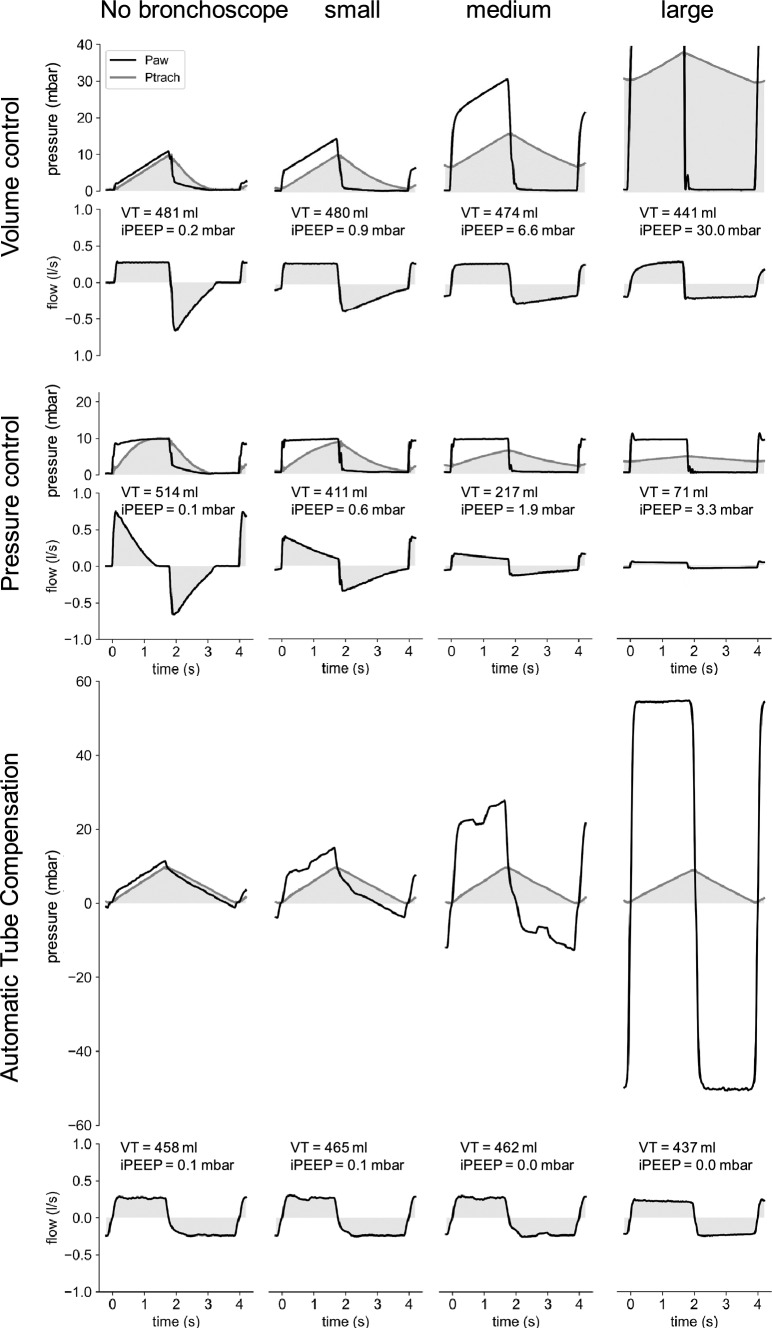
Airway pressure, tracheal pressure, and flow curves during volume-controlled ventilation (top), pressure-controlled ventilation without ATC (middle), and pressure-controlled ventilation with ATC (bottom) through a 7.0 mm tube without or with a small, medium or large bronchoscope. Pressure and flow are delivered by a commercial intensive care ventilator or an ATC prototype ventilator, with settings as described in Methods. The tube and artificial trachea are connected to an active lung simulator. Note that airway pressure exceeds the sensor’s 80 mbar range in VC mode for the large bronchoscope

The results of the numerical simulations closely agree with measurements of intrinsic PEEP obtained with an EVITA V600 ventilator and a lung simulator for different combinations of endotracheal tube and bronchoscope diameters (Fig. 4a). Therefore, the effects of an increased tube resistance during bronchoscopy can be accurately predicted , based on the ventilator settings, the patient’s respiratory mechanics, and the effective tube diameter. The pressure and flow traces (Fig. 5, top row) illustrate the dramatic airway pressure increase during inspiration and the progressive worsening of expiratory flow limitation with increasing bronchoscope diameter.

### Effects of tube resistance on tidal volume during pressure-controlled ventilation

The increase of the tube resistance during bronchoscopy not only causes flow limitation during expiration but also limits the inspiratory flow in pressure-controlled modes of ventilation (Fig. 5), leading to progressively decreasing tidal volumes for smaller effective tube diameters. Because of the decreasing tidal volumes, the intrinsic PEEP build-up is smaller in pressure-controlled compared to volume-controlled modes.

To estimate the intrinsic PEEP and tidal volume changes as a function of the effective tube diameter, we again perform a numerical simulation. The settings for inspiratory and expiratory time, valve resistance and respiratory system mechanics are the same as used above for simulating volume-controlled ventilation, except that we now supply a pressure support of 10 mbar with every breath. We find that for effective tube diameters above 7 mm, the resulting tidal volumes are 500 ml, without noticeable intrinsic PEEP build-up. Below 7 mm, tidal volume decreases, and intrinsic PEEP increases with smaller effective tube diameters (Fig. 4b). The results from the numerical simulations closely agree with measurements of intrinsic PEEP and tidal volumes obtained with a ventilator and a lung simulator for different combinations of endotracheal tube and bronchoscope diameters (Fig. 4b). The flow traces (Fig. 5) illustrate the progressive worsening of both inspiratory and expiratory flow limitation with increasing bronchoscope diameter.

### Effects of the respiratory system compliance on tidal volume and iPEEP

The increased resistance of the tube–bronchoscope combination increases the time constant of the expiratory flow, potentially leading to dynamic hyperinflation of the lungs when a constant tidal volume is enforced under volume-controlled ventilation. On one hand, an increased compliance and hence increased time constant exacerbates iPEEP. On the other hand, a higher compliance reduces the peak alveolar pressure according to Palv,peak = PEEP + V_T_/C, which tends to lower the end-inspiratory alveolar pressure and thereby reduces iPEEP. Numerical simulations demonstrate that these two opposing effects nearly cancel out, such that the build-up of iPEEP at small effective tube diameters is largely independent of the patient’s compliance under volume-controlled ventilation (Fig. SI 1a).

The effects of compliance changes are more pronounced under pressure-controlled ventilation. To maintain a desired tidal volume target V_T,target_, the inspiratory pressure support (PS) above PEEP is adjusted to lower values in patients with higher compliance, according to PS = V_T,target_/C. This also limits the intrinsic PEEP, which cannot exceed the level of pressure support. However, with increasing compliance, the time constant for filling and emptying the lungs is increased, which tends to increase the intrinsic PEEP and decrease the tidal volume. The latter effect dominates the response, and the effective diameter at which the tidal volume starts to decline shifts to higher values as the compliance increases (Fig. SI 1b).

### Effects of respiratory rate and I:E ratio on tidal volume and intrinsic PEEP

Increasing the respiratory rate is associated with increased minute ventilation and decreased time for inspiration and expiration. Not surprisingly, numerical simulations show higher intrinsic PEEP values under volume-controlled modes, and lower tidal volumes under pressure-controlled modes (Fig. SI 2). A prolonged expiration time, with I:E ratios of 1:3 or longer, can substantially decrease iPEEP in volume-controlled ventilation but also decreases the tidal volume in pressure-controlled ventilation (Fig. SI 3).

### Automatic compensation of tube resistance

Under the mode automatic tube compensation (ATC), the ventilator automatically increases or decreases the airway pressure to whatever value is needed for delivering a target tracheal pressure. This way, the flow-limiting effect of the tube resistance can be prevented. During expiration, it may be necessary to lower the airway pressure below PEEP, or even below atmospheric pressure, to maintain a positive tracheal pressure. Since commercial ventilators, to the best of our knowledge, are not equipped to supply sub-atmospheric pressure, we use a custom-built ventilator for testing if ATC is able to compensate for the increased tube resistance during bronchoscopy, and to maintain a desired tidal volume without intrinsic PEEP build-up.

When the ventilatory support under the mode ATC is delivered in the form of a linear tracheal target pressure ramp (Fig. 5), we find that the inspiratory airway pressure generated by the ATC ventilator resembles the airway pressure during volume-controlled ventilation (Fig. 5). Hence, regardless of the tube resistance, the tidal volume remains approximately constant, except when the airway pressure required to compensate for the tube resistance exceeds the maximum pressure that the ventilator can deliver (Fig. 5).

During expiration, the airway pressure required to compensate for the added tube resistance during bronchoscopy reaches sub-atmospheric levels, while the tracheal pressure remains equal or above PEEP. Lowering airway pressure during expiration prevents any intrinsic PEEP build-up. Taken together, our measurements confirm that the mode automatic tube compensation can effectively compensate for the added resistance during bronchoscopy and prevent both hypoventilation and intrinsic PEEP build-up.

## Discussion

Bronchoscopic procedures in mechanically ventilated intubated patients significantly increase airflow resistance and may compromise effective ventilation. The current study quantifies the flow resistance during bronchoscopy and provides insights into how it impacts ventilatory dynamics, in particular tidal volume and intrinsic positive end-expiratory pressure (iPEEP). Our results demonstrate that the effective diameter of the tube–bronchoscope combination is the sole parameter of practical relevance in defining flow resistance during bronchoscopy. Importantly, different combinations of endotracheal tubes and bronchoscopes yielding the same effective diameter will produce the exact same flow and pressure profiles, and hence the same ventilatory impairments, when ventilator settings are held constant.

Our data show that the resistance of an unobstructed endotracheal tube scales with the inner tube diameter D according to a power-law relationship with exponent -3.6. This finding is somewhat unexpected, as the gas flow in endotracheal tubes is predominantly turbulent [6], where a *D*^−5^ scaling would be expected [17]. The observed *D*^−3.6^ scaling for unobstructed tubes is likely explained by the fact that the endotracheal tube is only one component of the full flow path, which also includes the transition into a wider tracheal segment and a 90° swivel connector. These additional elements introduce significant pressure losses due to flow separation and curvature, which are less sensitive to tube diameter [6] and, therefore, reduce the apparent scaling exponent.

If a bronchoscope is inserted, the resistance becomes dominated by frictional losses in the tube. Experimentally, we find that the tube resistance scales with the effective tube diameter according to D_eff_^−−5^, as expected by theory. This stronger scaling exponent compared to an unobstructed tube likely results from the highly non-circular flow geometry introduced by the bronchoscope and from additional wall shear stresses along the bronchoscope surface, thereby enhancing frictional losses beyond those of a smooth-walled circular tube with equal cross-sectional area.

The pressure drop across a tube is well-captured by the Rohrer equation [16], which consists of a laminar and a turbulent flow component, k1 and k2 [16, 17]. We find that k2 tends to increase as k1 increases, hence k1 and k2 are co-variant. On average, our measurements show that k2 = 4 k1. This reduces the degrees of freedom and allows us to approximately describe the pressure drop across the tube with a single free parameter—the effective tube diameter—according to

3 $${\Delta \text{p}}_{\text{ETT}}=\text{k}1{\left(\frac{{\text{D}}_{\text{eff}}}{{\text{D}}_{0}}\right)}^{\upbeta }\left(\dot{\text{V}}+\text{sign}\left(\dot{\text{V}}\right)4{\left(\frac{\dot{\text{V}}}{{\dot{\text{V}}}_{0}}\right)}^{2}\right)$$

with k1 = 0.72 mbar, and exponent β = −3.6 for an unobstructed tube (without bronchoscope), and β  = −5 for a tube with inserted bronchoscope. D_0_ is a reference diameter set to 10 mm. This relationship holds for any tube–bronchoscope combination, for tube diameters between 6.0 and 9.0 mm, and bronchoscope diameters between 3.8 and 5.9 mm. The mean absolute percentage error of Eq. 3 for describing the resistance of unobstructed tubes is 2.4% , and 10.6% for describing the resistance of tubes with inserted bronchoscope.

This universal relationship with only one free parameter, D_eff_, allows us to predict the effects of different endotracheal tube–bronchoscope combinations on the flow and pressure profile during mechanical ventilation, and to compute the resulting intrinsic PEEP and tidal volumes (Fig. 4). Because of the nonlinearity of the tube resistance, this computation requires a numerical simulation. We have confirmed that the predictions from the numerical simulation closely agree with measurements obtained with an intensive care ventilator and a lung simulator, for different tube–bronchoscope combinations.

Our lung simulator and numerical model represent the respiratory system as a single compartment with constant compliance (C_rs_), constant airway resistance (R_aw_), and flow-dependent endotracheal tube resistance (R_ETT_). Thus, phenomena such as expiratory airway closure, lung collapse and recruitment, volume-dependent compliance and resistance, or secretions are not considered. In addition, we tested only a single-size, rigid-walled artificial trachea to mimic flow separation at the endotracheal tube tip. Despite these simplifications, the model approximates patient respiratory mechanics reasonably well under standard clinical conditions [6, 8].

This study identifies the effective diameter, computed according to $\sqrt{{{\text{D}}_{\text{tube}}}^{2}-{{\text{D}}_{\text{scope}}}^{2}}$, as the crucial parameter that determines the degree of ventilation impairments. Conclusions from earlier studies that the endotracheal tube diameter should be at least 2 mm larger than the diameter of the bronchoscope to maintain volume delivery and minimize the development of auto-PEEP [12, 14] are, according to our findings, too optimistic, especially in smaller tubes. For 7.0 or 8.0 mm tubes, for example, a bronchoscope that is 2 mm smaller will result in effective diameters of 4.9 or 5.3 mm, respectively. In both cases, this will lead to significant iPEEP build-up and decreased tidal volume even in a patient with a low respiratory rate and minute ventilation (Fig. 4).

The strong power-law increase of tube resistance with effective diameter implies that there is no universally applicable threshold for a safe effective diameter. For example, even a 7 mm tube without a bronchoscope can cause substantial iPEEP and a decline in tidal volume in patients requiring high minute ventilation. We, therefore, recommend using the provided simulation tool to anticipate ventilation impairments and to guide case-specific decisions at the bedside. The clinical decision to select an appropriate bronchoscope diameter must necessarily integrate additional patient-specific factors beyond the effective diameter, such as airway anatomy, pathology, or the rheology of secretions that block the airways.

The decision to use either volume-controlled or pressure-controlled ventilation during bronchoscopy should be based, among other factors, on weighing the risk of excessive iPEEP against the risk of reduced ventilation. If volume-controlled ventilation is chosen, which has the advantage of delivering the selected tidal volume regardless of tube resistance, we recommend prolonging the expiration time and aiming for higher I:E ratios. In this mode, it is important to monitor the end-expiratory flow to detect intrinsic PEEP build-up. A lower bound for intrinsic PEEP can be estimated at the bedside from the pressure loss across the tube at end-expiration [18], calculated according to Eq. 3. It must be kept in mind, however, that in clinical practice the flow resistance of endotracheal tubes can be considerably higher, e.g., due to secretions, than that of the clean tubes studied here [19]. Under pressure-controlled ventilation, the main detrimental effect of bronchoscopy—a decline in tidal volume—is more easily detectable and can be counteracted by increasing pressure support, although this will also increase intrinsic PEEP.

This study demonstrates that the added tube resistance during bronchoscopy can be effectively compensated using full (inspiratory and expiratory) automatic tube compensation (ATC), as opposed to partial ATC as implemented in commercial ventilators [20]. We further show that full ATC can prevent both the reduction in tidal volume and the build-up of intrinsic PEEP. However, this may require lowering airway pressure below atmospheric levels—a function that, to our knowledge, is not available in current commercial ventilators. Our findings provide a compelling rationale to develop ventilators capable of sub-atmospheric expiratory pressure delivery and to incorporate resistance profiles for specific tube–bronchoscope combinations.

## Conclusions

The effective diameter provides a practical basis for predicting ventilatory impairments during bronchoscopy. To identify strategies that minimize impairments, we provide a freely available quantitative framework at https://fabrylab.github.io/Bronchoscopy/ for estimating iPEEP and tidal volume as functions of effective diameter, ventilator settings, and patient respiratory mechanics. Finally, we demonstrate proof of principle that automatic tube compensation can prevent ventilatory impairments during bronchoscopy.

## Supplementary Information

Supplementary file 1.

## Data Availability

Raw data can be obtained from the corresponding author upon request. The program for predicting ventilation impairments is freely available under MIT license and can be downloaded via the open access repository (https://fabrylab.github.io/Bronchoscopy/).
