# Supplementary material for: Prediction and prevention of ventilation impairments during bronchoscopy
Source: Intensive Care Med Exp. 2025 Dec 17;13:130. doi: 10.1186/s40635-025-00846-5 (PMC12712257; doi:10.1186/s40635-025-00846-5)
Supplement: Supplementary file 1 [file 40635_2025_846_MOESM1_ESM.docx]

Supplementary Information

**Prediction and Prevention of Ventilation Impairments During Bronchoscopy**

Ben Fabry, Navid Bonakdar, Christian Kuster, Johannes Bartl, Frederick Krischke, Roland Francis

**Table S1**: Fit parameters of Eq. 1 for describing the pressure drop across the tube for different tube-bronchoscope combinations

| ETT ø | bronchoscope ø | Deff | k1 in,ex | k2 in | k2 ex | rms | MAPE |
| --- | --- | --- | --- | --- | --- | --- | --- |
| mm | mm | mm | mbar | mbar | mbar | mbar | % |
| 6.0 | 0.0 | 6.00 | 2.49 | 20.50 | 21.43 | 0.59 | 1.20 |
| 6.5 | 0.0 | 6.50 | 1.59 | 14.53 | 14.75 | 0.42 | 1.30 |
| 7.0 | 0.0 | 7.00 | 1.19 | 12.44 | 10.91 | 0.32 | 1.14 |
| 7.5 | 0.0 | 7.50 | 0.69 | 9.60 | 9.45 | 0.32 | 1.62 |
| 8.0 | 0.0 | 8.00 | 0.47 | 7.32 | 7.30 | 0.27 | 1.89 |
| 8.5 | 0.0 | 8.50 | 0.90 | 5.76 | 5.53 | 0.28 | 1.77 |
| 9.0 | 0.0 | 9.00 | 0.45 | 4.67 | 4.69 | 0.22 | 1.62 |
| 6.0 | 3.8 | 4.64 | 24.39 | 107.17 | 111.22 | 0.50 | 1.91 |
| 6.5 | 3.8 | 5.27 | 10.64 | 56.61 | 58.52 | 0.71 | 1.64 |
| 7.0 | 3.8 | 5.88 | 8.84 | 34.47 | 31.72 | 0.57 | 2.39 |
| 7.5 | 3.8 | 6.47 | 7.12 | 22.82 | 21.32 | 0.75 | 4.30 |
| 8.0 | 3.8 | 7.04 | 3.19 | 16.57 | 15.65 | 0.55 | 3.29 |
| 8.5 | 3.8 | 7.60 | 2.93 | 11.65 | 12.10 | 0.47 | 3.11 |
| 9.0 | 3.8 | 8.16 | 1.11 | 8.86 | 8.91 | 0.30 | 2.52 |
| 6.5 | 5.0 | 4.15 | 56.30 | 206.25 | 225.41 | 0.60 | 2.61 |
| 7.0 | 5.0 | 4.90 | 30.17 | 106.40 | 102.36 | 0.60 | 2.21 |
| 7.5 | 5.0 | 5.59 | 13.65 | 56.64 | 56.67 | 0.69 | 1.29 |
| 8.0 | 5.0 | 6.24 | 8.09 | 30.52 | 29.93 | 0.57 | 1.70 |
| 8.5 | 5.0 | 6.87 | 6.25 | 20.81 | 20.97 | 0.58 | 2.62 |
| 9.0 | 5.0 | 7.48 | 2.86 | 13.36 | 13.56 | 0.46 | 2.03 |
| 7.0 | 5.9 | 3.77 | 109.74 | 464.40 | 475.09 | 0.82 | 1.45 |
| 7.5 | 5.9 | 4.63 | 45.30 | 137.78 | 139.33 | 0.54 | 3.76 |
| 8.0 | 5.9 | 5.40 | 14.49 | 68.87 | 72.43 | 0.75 | 2.20 |
| 8.5 | 5.9 | 6.12 | 12.84 | 36.74 | 35.92 | 0.58 | 3.45 |
| 9.0 | 5.9 | 6.80 | 5.61 | 20.66 | 19.80 | 0.64 | 1.61 |

Deff: effective tube diameter according to Eq. 2

rms: root mean squared error between measured and fitted pressure drop values across the tube (see Fig. 2), excluding all pressure values < 1 mbar

MAPE: mean absolute percentage error between measured and fitted pressure drop values across the tube (see Fig. 2), excluding all pressure values < 1 mbar

**
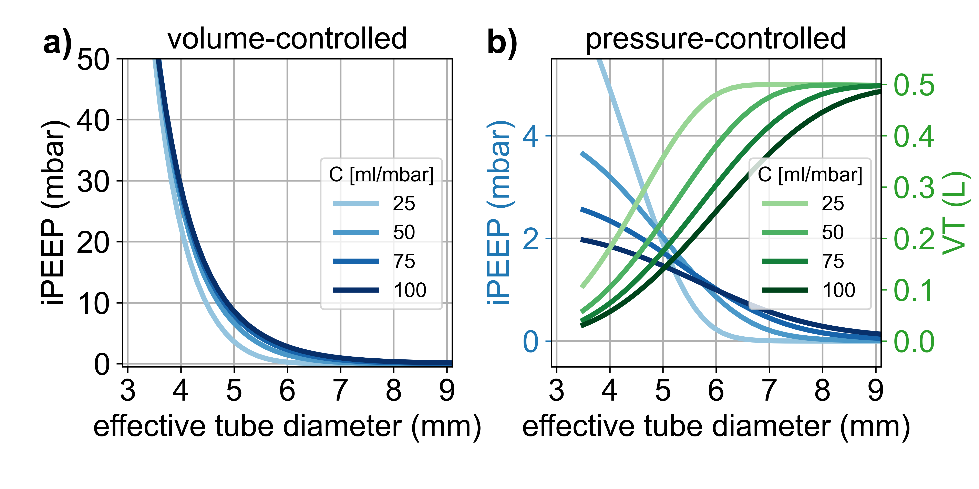
**

**Fig. SI 1**. Influence of respiratory system compliance (C = 25, 50, 75 and 100 ml/mbar) on iPEEP and tidal volume. **a)** Intrinsic PEEP build-up during volume-controlled mechanical ventilation, for V_T_ = 500 ml, rr = 15/min and t_ex_ = 2.2 s, as a function of the effective tube diameter. **b)** Intrinsic PEEP build-up (blue) and tidal volume changes (green) during pressure-controlled mechanical ventilation for a pressure support (PS) of 10 mbar, RR = 15/min and T_ex_ = 2.2 s, as a function of the effective tube diameter. The PS is adjusted according to PS = 500 ml / C so that the maximum tidal volume is 500 ml regardless of compliance. In both graphs, the lines show the results from a numerical simulation based on the resistances of tubes with inserted bronchoscope taken from Eq. 3, for a patient with R_aw_ = 2 mbar/(L/s).

**
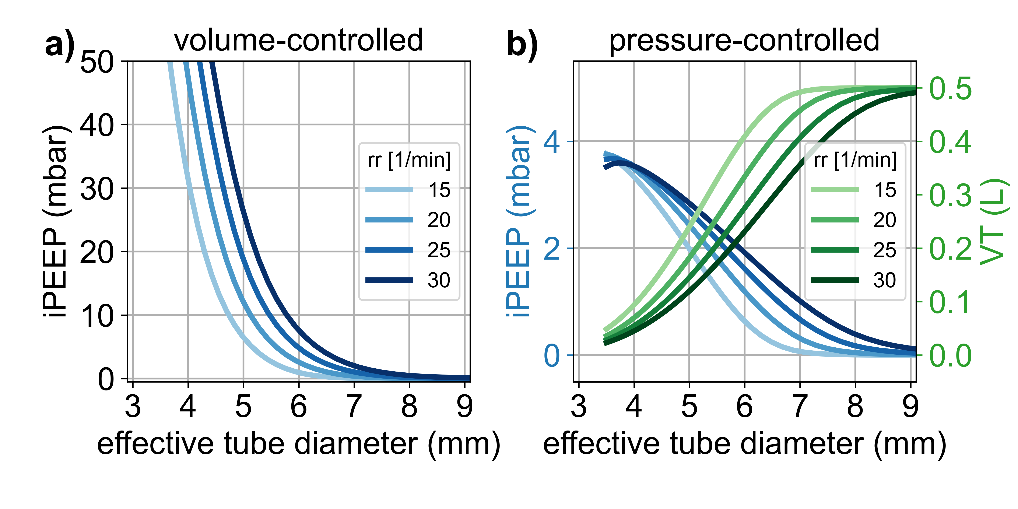
**

**Fig. SI 2**. Influence of respiratory rate (RR = 15, 20, 25 and 30 min^-1^) on iPEEP and tidal volume. I:E ratio is set to 1:1.2 in all cases. **a)** Intrinsic PEEP build-up during volume-controlled mechanical ventilation, for V_T_ = 500 ml, as a function of the effective tube diameter. **b)** Intrinsic PEEP build-up (blue) and tidal volume changes (green) during pressure-controlled mechanical ventilation for a pressure support of 10 mbar, as a function of the effective tube diameter. In both graphs, the lines show the results from a numerical simulation based on the resistances of tubes with inserted bronchoscope taken from Eq. 3, for a patient with C = 50 ml/mbar, R_aw_ = 2 mbar/(L/s).

**
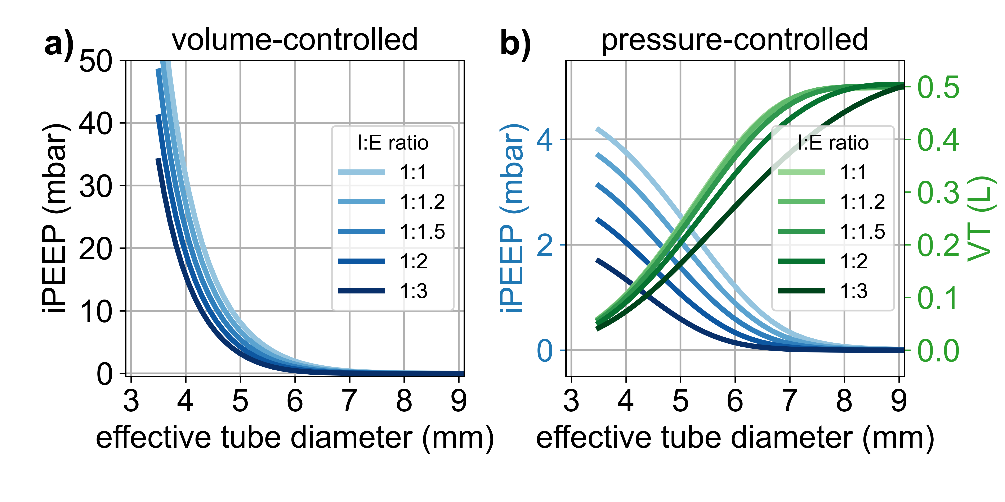
**

**Fig. SI 3**. Influence of I:E ratio on iPEEP and tidal volume. Respiratory rate is set to 15 breaths per minute in all cases. **a)** Intrinsic PEEP build-up during volume-controlled mechanical ventilation, for V_T_ = 500 ml, as a function of the effective tube diameter. **b)** Intrinsic PEEP build-up (blue) and tidal volume changes (green) during pressure-controlled mechanical ventilation for a pressure support of 10 mbar, as a function of the effective tube diameter. In both graphs, the lines show the results from a numerical simulation based on the resistances of tubes with inserted bronchoscope taken from Eq. 3, for a patient with C = 50 ml/mbar, R_aw_ = 2 mbar/(L/s).

**Technical description of the lung simulator**

The lung simulator used in this study is custom-built. It consists of a piston pump with a maximum tidal volume of 1.8 L and is driven by a stepper motor. It can simulate active breathing as well as the passive mechanics of the respiratory system according to a single-compartment linear model with a constant airway resistance and a constant compliance (or elastance).

In our experimental setup (see Fig. 1 of the main text), the lung simulator is placed behind the artificial trachea and the endotracheal tube. The stepper motor of the lung simulator is controlled such that the tracheal pressure P_trach_ (measured at the connection between the artificial trachea and the tubing connecting to the lung simulator) satisfies the equation of motion:

$P_{trach}= {\dot{V}R}_{aw}+\frac{V}{C_{rs}}+P_{mus}$ Eq. 1

with airway resistance R_aw_, respiratory system’s compliance C_rs_, gas flow$\dot{V}$, lung volume V above the functional residual capacity (FRC), and muscle pressure P_mus_.

P_trach_ is measured with a piezo-resistive pressure transducer (HCS-series Honeywell (USA) sensors, ±80 mbar range) at 250 Hz. Gas flow ($\dot{V}$) is not directly measured but inferred from piston motion (Q, in L/s) and the rate of pressure change (dP_trach_/dt), accounting for the compressibility of air in the total volume (tubing volume + 0.3 L residual piston volume + added piston volume V_piston_) (Eq. 2).

We also connect a 20 L glass balloon filled loosely with steel wool to the tubing between the lung simulator and the trachea. This added gas reservoir has the purpose of dampening rapid pressure fluctuations produced by discrete stepper motor movements. The gas reservoir sets a lower limit of 0.02 L/mbar to the compliance that can be simulated. Assuming isothermal conditions inside the reservoir, the air compressibility is taken as c=0.1% per mbar, and the gas flow is calculated according to

$\dot{V}=Q+c\left( V_{piston}+0.3L+20L \right)\frac{dP_{trach}}{dt}$ Eq. 2

The piston volume is obtained by summing the stepper motor increments, converted to liters, ($V_{piston}=\Sigma Q$). Lung volume above FRC is then the sum of the piston volume and the volume required to compress the air in the system, according to

$V=V_{piston}+c\left( V_{piston}+0.3L+20L \right)P_{trach}$ Eq. 3

Combining Eqs. 1-3 yields

$Q=\frac{P_{trach}-P_{mus}-\frac{V}{C_{rs}}}{R_{aw}}-c\left( V_{piston}+0.3L+20L \right)\frac{dP_{trach}}{dt}$ Eq. 4

Q and P_trach​_ are low-pass filtered (first-order, cutoff 25 Hz) to prevent control instability. In its current implementation of the control software, the second term on the right-hand side of Eq. 4 can induce oscillations and is therefore ignored. Q is then converted to number of steps that are being sent to the stepper motor. This control equation ensures that the tracheal pressure follows the prescribed equation of motion (Eq. 1) at each 4 ms update interval.

**Fig. SI 4**. Working principle of the lung simulator.

All measurements and real-time control are performed on a Teensy 4.0 microcontroller (PJRC, Sherwood, OR, USA, based on an NXP i.MX RT1062 ARM Cortex-M7 processor) at a control rate of 250 Hz. Stepper pulses are amplified by a stepper motor driver (DMC542, Changzhou ACT Motor, China) and transmitted to the stepper motor, which actuates a spindle-nut mechanism coupled to the piston.

The measured P_trach_​ and the computed gas flow (Eq. 2) are transmitted via RS-232 to a PC. A Python-based graphical interface running on the PC displays P_trach_​ and flow waveforms, and allows the user to adjust compliance, resistance, muscle pressure amplitude, and breathing frequency. Settings are communicated back to the microcontroller via RS-232.


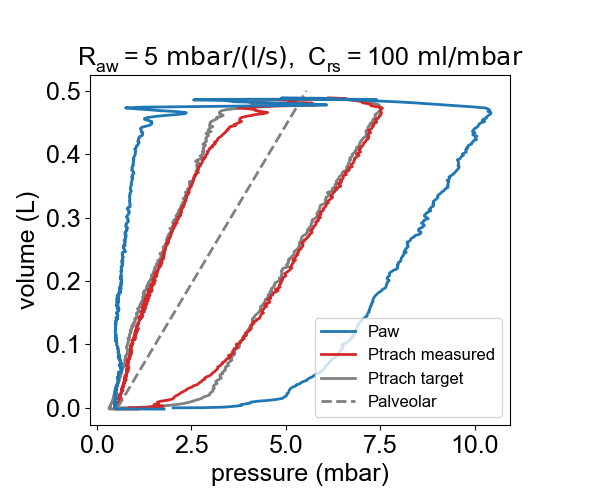

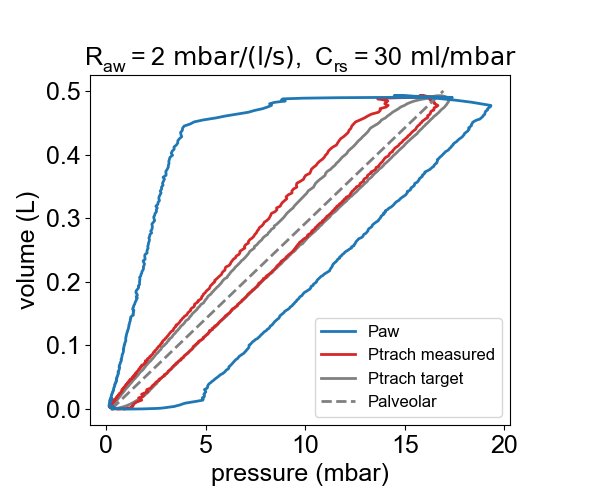


**Fig. SI 5**. Example pressure-volume loops, measured under volume-controlled ventilation (t_in_ = 1.2 s, t_ex_ = 2.8 s, inspiratory flow = 0.42 L/s) through a 7 mm endotracheal tube, delivered by an Evita V600 ventilator. PEEP was set to 0 mbar, but the effectively measured PEEP was 0.5 mbar. The solid gray lines represent the target tracheal pressure required to satisfy the equation of motion for the R_aw_ and C_rs_ parameters specified in the figure title. Deviations between the target tracheal pressure and the measured tracheal pressure (red), especially during rapid flow changes at the beginning of inspiration and expiration, are due to stepper-motor control delays.


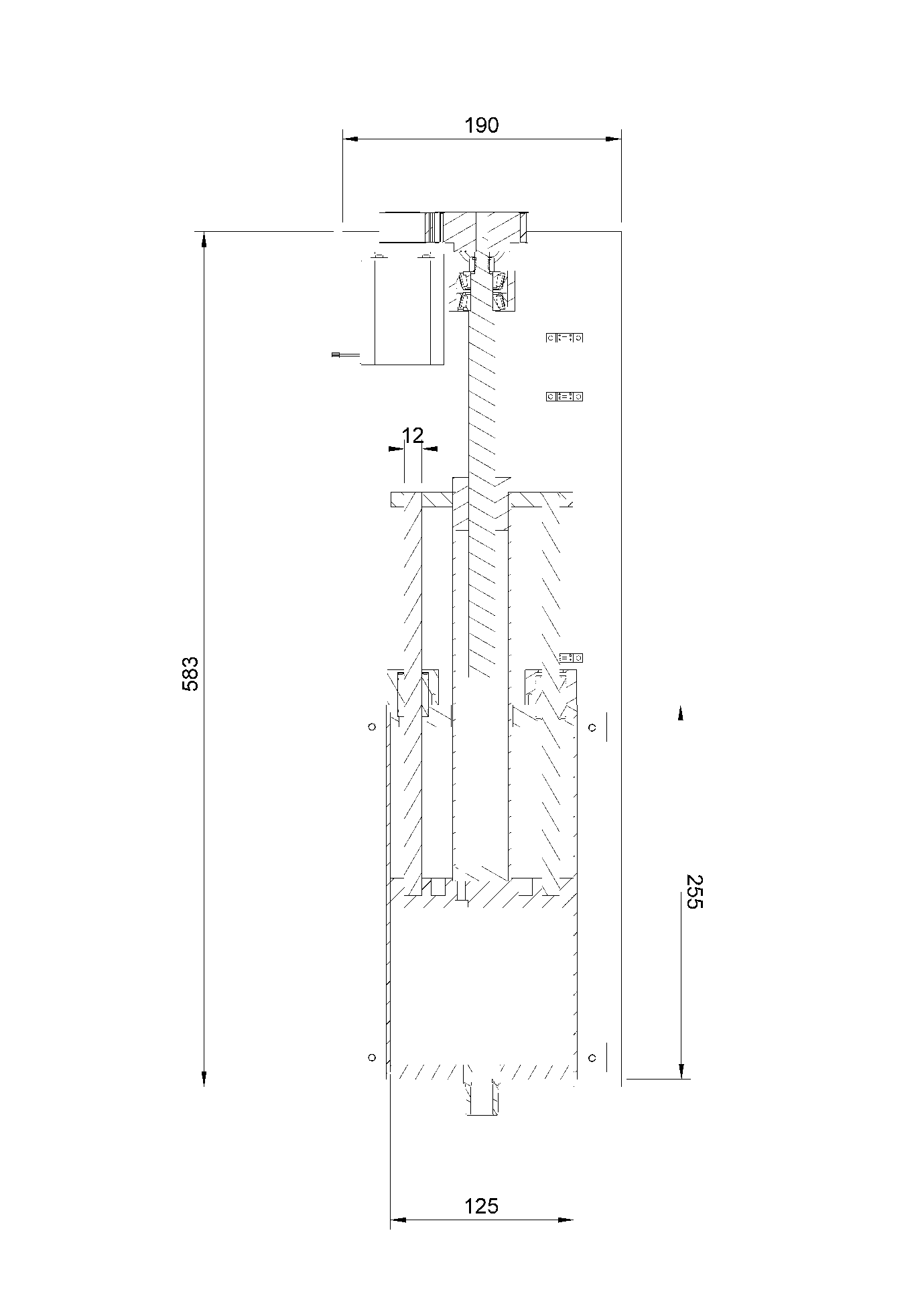


**Fig. SI 6.** Technical drawing of the lung simulator.


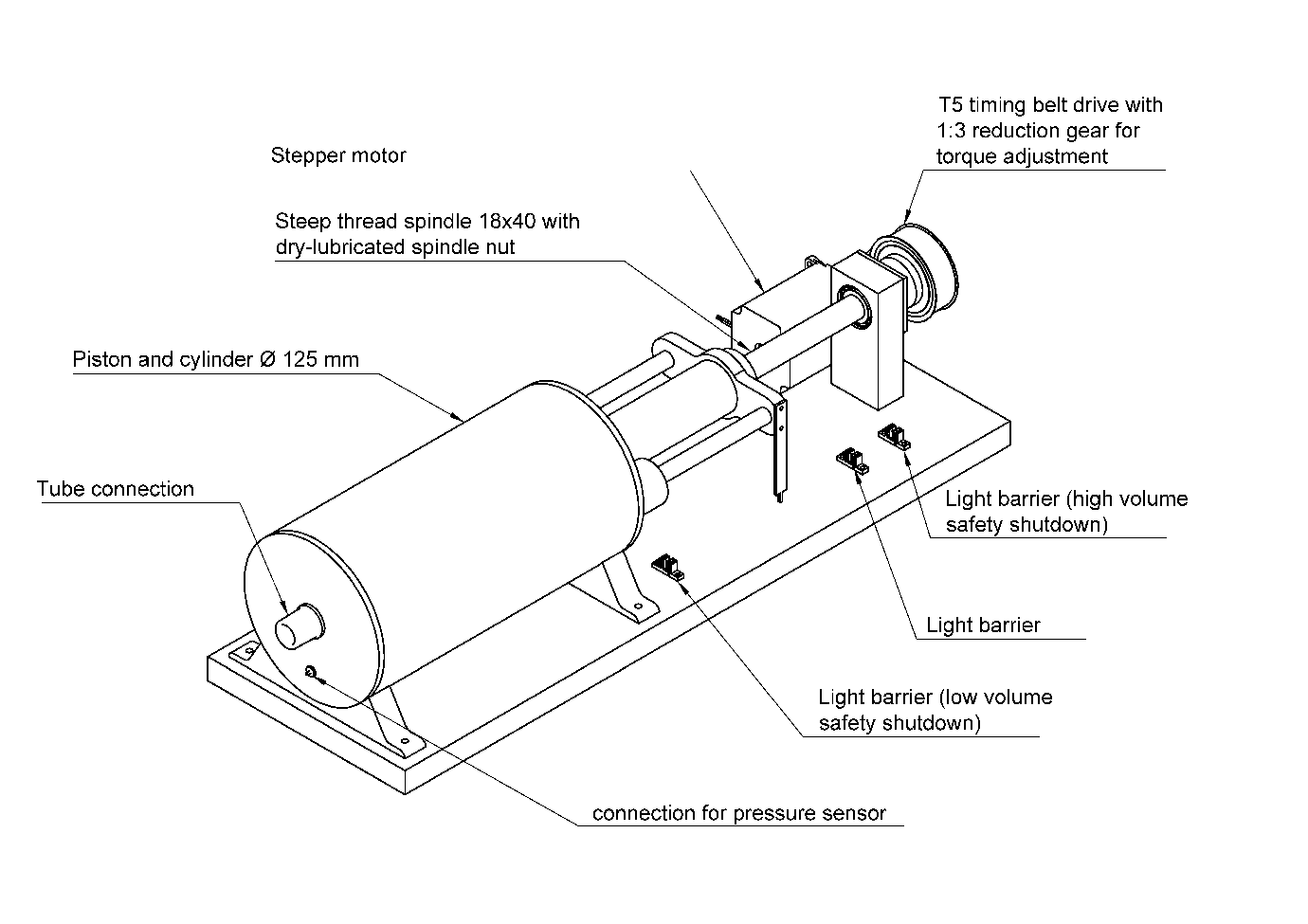


**Fig. SI 7.** Technical illustration of the lung simulator.
